# Association between Metformin Use and Coronary Artery Calcification in Type 2 Diabetic Patients

**DOI:** 10.1155/2019/9484717

**Published:** 2019-05-05

**Authors:** Yi Lu, Yidong Wang, Ting Weng, Zexin Chen, Xiujuan Sun, Jia Wei, Zhejun Cai, Meixiang Xiang

**Affiliations:** ^1^Department of Cardiology, Second Affiliated Hospital of Zhejiang University School of Medicine, Cardiovascular Key Lab of Zhejiang Province, #88 Jiefang Road, Hangzhou, Zhejiang 310009, China; ^2^Department of Clinical Epidemiology and Biostatistics, Second Affiliated Hospital, Zhejiang University School of Medicine, Hangzhou, China; ^3^Department of Radiology, Second Affiliated Hospital, Zhejiang University School of Medicine, Hangzhou, China; ^4^Department of Urology, Children's Hospital, Zhejiang University School of Medicine, Hangzhou, China; ^5^The Key Laboratory of Cardiovascular Remodeling and Function Research, Chinese Ministry of Education and Chinese Ministry of Health, and The State and Shandong Province Joint Key Laboratory of Translational Cardiovascular Medicine, Qilu Hospital of Shandong University, Jinan, China

## Abstract

**Objectives:**

Type 2 diabetes mellitus (T2DM) is associated with coronary artery calcification (CAC) which is an independent risk factor for cardiovascular events. Metformin is the first-line antidiabetic medication. We aimed to investigate the association between metformin use and CAC.

**Methods:**

We included 369 patients with T2DM in this cross-sectional study. CAC scores, clinical characteristics, and antidiabetic drug prescription information of the patients were acquired. Baseline parameters were balanced for metformin and nonmetformin users using the propensity score matching (PSM) strategy.

**Results:**

Among the 369 subjects who met our inclusion criteria, 288 subjects were included for further analysis after PSM. Metformin prescription rather than other antidiabetic medications was related to lower CAC scores (OR [95% CI] = 0.55 [0.34–0.90]; *P* = 0.018). Further multivariable logistic regression analysis demonstrated that metformin was negatively associated with CAC severity (OR [95% CI] = 0.58 [0.34–0.99]; *P* = 0.048), which was independent of age, BMI, eGFR, gender, cigarette smoking, duration of diabetes, hypertension, statin prescription, and number of nonmetformin antidiabetic agents. A subgroup analysis revealed a significant association between metformin and CAC scores in smokers (OR [95% CI] = 0.38 [0.16–0.93]; *P* = 0.035), but the association was not observed in never-smokers (OR [95% CI] = 0.72 [0.34–1.51]; *P* = 0.383).

**Conclusions:**

Metformin usage was independently associated with lower CAC scores in T2DM patients. The negative correlation between CAC scores and metformin was most prominent in patients with a history of cigarette smoking.

## 1. Introduction

During the past decades, compelling evidence has demonstrated that coronary artery calcification (CAC) is an independent risk factor of cardiovascular events. Moreover, CAC is a challenge for percutaneous coronary intervention (PCI) and is linked with increased post-PCI events. However, there is no clinically proved therapy for vascular calcification [1]. Type 2 diabetes mellitus (T2DM) is deemed as a coronary artery disease (CAD) equivalent [2], which doubles or even triples the CAD incidence [3]. Moreover, T2DM patients tend to suffer from more calcified and diffuse coronary artery lesions, while having a blunted appreciation of ischemic episodes [4].

Imaging by computed tomography (CT) reveals that T2DM-affected individuals have extensive calcification of their vascular beds, which is reported as the CAC scores, reflecting significant cardiovascular disease burden [5, 6]. The CAC score has independent added value beyond traditional risk factors in predicting the outcome of major cardiovascular events, especially in asymptomatic patients [7]. In patients with an intermediate Framingham risk score, CAC scores less than 99, between 100 and 399, and more than 400 are related to 0.4%, 1.3%, and 2.4% of annual CAD death, respectively [8].

As the first-line antidiabetic therapy, recent studies indicate that metformin has highlighted effect on alleviating vascular calcification. We and other groups reported that metformin prevents vascular calcification via AMP-activated protein kinase (AMPK) activation [9, 10], and we identify that metformin prevents atherosclerotic calcification in mice [10]. Moreover, recent clinical data showed that metformin prescription was independently associated with a decreased level of lower-limb arterial calcification [11]. Thus, it is reasonable to hypothesize that metformin therapy may be associated with lower levels of CAC severity in T2DM patients. We therefore performed a cross-sectional study in a population of asymptomatic T2DM patients to evaluate the association between metformin use and CAC scores.

## 2. Materials and Methods

### 2.1. Study Design

This is a cross-sectional study conducted among in-hospital T2DM patients who underwent coronary artery CT for preoperative screening between June 1^st^, 2016, and May 31^st^, 2017, in the Second Affiliated Hospital, Zhejiang University School of Medicine. Those patients were candidates for noncardiac surgery including hip/knee replacement, lumbar surgery, radical resection of pulmonary carcinoma, and cerebral artery aneurysm intervention. Main inclusion criteria were (1) diagnosed as T2DM and took antidiabetic drugs regularly for at least 3 months and (2) antidiabetic drug prescription remained unchanged for the last 3 months. Main exclusion criteria were (1) a history of CAD or PCI or coronary artery bypass grafting or clinical presentation of CAD like chest pain and shortness of breath; (2) type 1 diabetes mellitus; and (3) glomerular filtration rate ≤ 30 mL/min measured by the CKD-EPI equation [12]. The study was conducted on the grounds of the Declaration of Helsinki and approved by the Ethics Committee of the Second Affiliated Hospital, Zhejiang University School of Medicine.

### 2.2. CAC Quantification

Coronary artery CT images were acquired with Siemens SOMATOM definition flash CT. A radiologist who was blinded to patients reread all CT scans and reassessed their CAC scores. CAC scores were calculated with the Agatston method [13], i.e., by multiplying the area with a density above 130 Hounsfield units (Hu) by a factor reflecting the maximum attenuation.

### 2.3. Data Collection

We collected fasting blood data within 7 days before the coronary artery CT scan, in avoidance of the interference of contrast-induced nephropathy. This work was accomplished by one clinician, who was blinded to patients' medical history. Hemoglobin A1c (HbA1c), serum creatinine, fasting glucose, calcium, phosphate, low-density lipoprotein cholesterol (LDL-C), high-density lipoprotein cholesterol (HDL-C), and triglyceride (TG) results were collected. The CKD-EPI equation was adopted for estimating the glomerular filtration rate (eGFR).

### 2.4. Statistical Analyses

Data were presented as means ± standard deviation (SD) or median (25^th^ and 75^th^ percentiles) for continuous variables, as appropriate. Data were expressed as the number (percentage) for qualitative variables. Data were categorized into metformin and nonmetformin users. Major imbalances were found in age, BMI, history of hypertension, eGFR, and glucosidase inhibitor usage between metformin and nonmetformin users. Those imbalanced factors were used to perform propensity score matching (PSM) for patients in two groups. A multivariable logistic regression model including these variables was applied. Matching was performed using the nearest neighbor matching, with a default caliper of 0.1. The CAC score = 100 Agatston units was set as the cutoff point in accordance with the risk classification in ACCF/AHA 2007. The *χ* ^2^ test was used to compare categorical variables, and Student's *t*-test or the Mann–Whitney test was used for continuous variables between the two groups. The association between antidiabetic drugs and CAC scores was analyzed by univariate logistic regression. To assess the independence of this association, we performed a multivariable logistic regression that included both variables identified in univariate analyses and relevant clinical variables or demographic factors (age, BMI, gender, eGFR, duration of diabetes and hypertension, and cigarette smoking). Furthermore, we did a subgroup analysis among male and female patients, which demonstrated that there was no gender difference. Another subgroup analysis among patients with or without a history of smoking was processed. Multivariable logistic regression with the same adjustments was used in the subgroup analysis. All statistical analyses were performed using IBM SPSS Statistics for Windows, version 22.0 (IBM, Armonk, New York).

## 3. Results

### 3.1. Demographic and Clinical Characteristics

**Figure 1:**
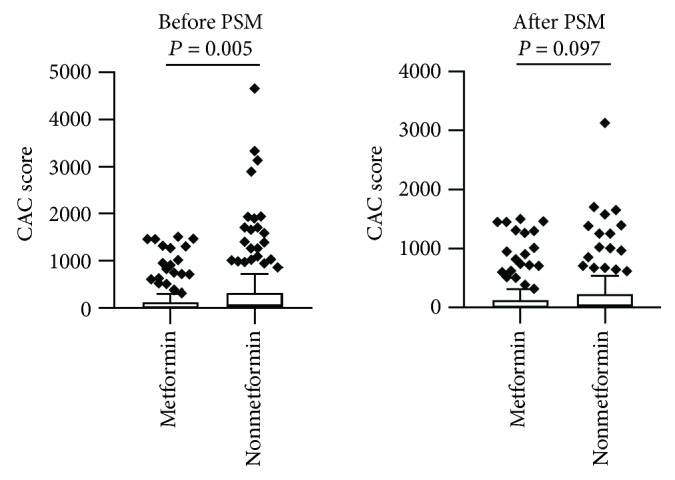
The comparison of CAC scores between patients treated with or without metformin. The Mann–Whitney test was used for the comparison between patients treated with or without metformin. The median CAC scores were 8.05 (0, 124.6) and 61.6 (0, 319.6), respectively (*P* = 0.005), before propensity score matching (PSM). And median CAC scores were 8.05 (0, 124.6) and 37.00 (0.00, 220.50) (*P* = 0.097) after matching.

Among the 656 subjects, we excluded 287 subjects who did not meet our inclusion criteria. The remaining 369 subjects were divided into the metformin group (*n* = 150) and the nonmetformin group (*n* = 219). The median CAC score in the metformin group was 8.05 (0-124.6), and the score was almost octupled in the nonmetformin group (61.6 (0-319.6), *P* = 0.005) (Figure 1). Major imbalances were spotted in age (*P* < 0.001), BMI (*P* = 0.005), history of hypertension (*P* = 0.011), eGFR (*P* < 0.001), and glucosidase inhibitor usage (*P* = 0.012) between metformin and nonmetformin users. We performed PSM of the imbalanced factors and excluded another 81 unmatched subjects. Demographic and clinical characteristics of metformin and nonmetformin users before and after PSM were presented in Table 1, and the flow chart of exclusion was presented in Supplemental Figure 1. The median CAC score in the metformin group after PSM was 8.05 (0-124.6), and the score in the nonmetformin group after PSM was 37.00 (0.00, 220.50), but *P* value is marginal (0.097) (Table 1, Figure 1).

In accordance with the risk classification in ACCF/AHA 2007, we categorized the subjects as CAC < 100 and CAC ≥ 100 to clarify the baseline differences between subjects with mild and moderate calcification (Table 2). Subjects in the higher CAC score group were significantly older and had longer history of hypertension. The higher CAC score was also related to lower eGFR. There were no significant intergroup differences for BMI, gender, smoking status, or relevant laboratory index including serum calcium, phosphate, fasting glucose, LDL-C, HDL-C, or TG. The data of HbA1c of 114 subjects were missing, but there was no intergroup difference in the remaining 255 subjects. Another interesting finding was that a higher percentage of subjects underwent statins or antiplatelet therapies, like aspirin and clopidogrel in the group with higher CAC scores (*P* < 0.05). The associations remained unchanged after PSM.

Notably, the CAC scores of 146 subjects were 0. In order to investigate the characteristics of subjects with CAC = 0, we classified subjects into CAC = 0, 0 < CAC < 100, and CAC ≥ 100 (Supplemental Table 1). Metformin was used in 46.58%, 49.37%, and 30.07% subjects in CAC = 0, 0 < CAC < 100, and CAC ≥ 100 groups, respectively. The intergroup difference was also significant (*P* = 0.004).

### 3.2. Association between Antidiabetic Therapy and CAC Severity

As per Table 2, metformin usage had a significant difference between CAC < 100 and ≥100 groups. Interestingly, 47.3% of subjects were treated with metformin in the CAC < 100 group, while the proportion was 30.1% in the ≥100 group, indicating that metformin usage corresponded to the lower CAC scores. Subjects in the groups of CAC < 100 and ≥100, respectively, took 1.16 ± 0.69 and 1.26 ± 0.76 kinds of nonmetformin antidiabetic drugs, which were not significantly different between the two groups (*P* = 0.230). The estimation association remained unchanged after PSM (Table 2).

**Figure 2:**
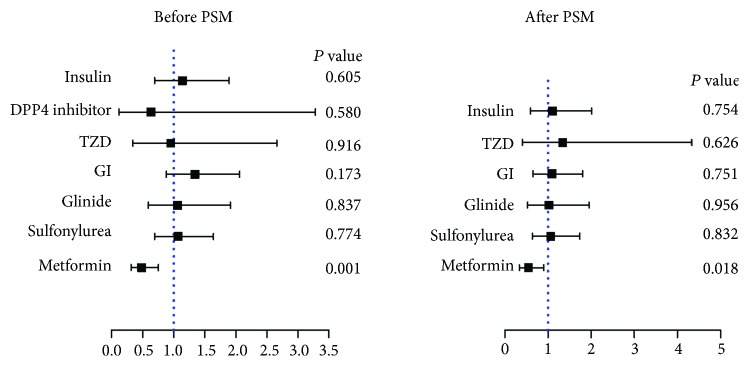
The association between metformin and other antidiabetic medicines and CAC scores. Metformin was negatively related to the CAC score before (OR [95% CI] = 0.48 [0.31–0.75]; *P* = 0.001) and after propensity score matching (OR [95% CI] = 0.55 [0.34–0.90]; *P* = 0.018). BMI: body mass index; DM: diabetes mellitus; HTN: hypertension; eGFR: estimated glomerular filtration rate; NMA: nonmetformin antidiabetic agents; PSM: propensity score match. Since the DPP4 inhibitor user was 0 among subjects with CAC ≥ 100 after propensity score matching, we omitted this data accordingly.

Univariate logistic regression was further applied, with CAC score = 100 as the cutoff point. The result confirmed that metformin prescription was related to lower CAC scores (OR [95% CI] = 0.55 [0.34–0.90]; *P* = 0.018). In contrast, the usage of other antidiabetic medicines did not affect the CAC scores (Figure 2, Supplemental Table 2).

### 3.3. Independent Association between Metformin and CAC Severity

**Figure 3:**
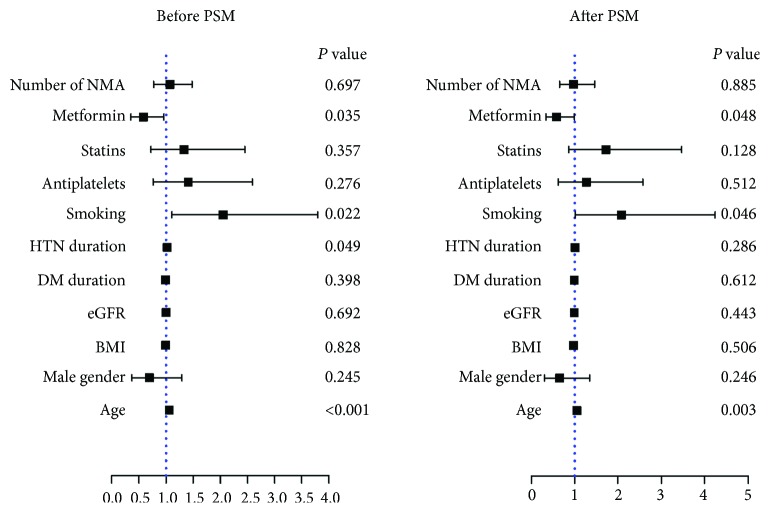
The independent association of metformin and other factors with CAC scores. The multivariable logistic regression analysis demonstrated that metformin was negatively associated with CAC severity before (OR [95% CI] = 0.58 [0.35–0.96]; *P* = 0.035) and after propensity score matching (OR [95% CI] = 0.58 [0.34–0.99]; *P* = 0.048), while smoking (OR [95% CI] = 2.08 [1.01–4.24]; *P* = 0.046) and age (OR [95% CI] = 1.05 [1.02–1.09]; *P* = 0.003) were positively associated with CAC severity. BMI: body mass index; DM: diabetes mellitus; HTN: hypertension; eGFR: estimated glomerular filtration rate; NMA: nonmetformin antidiabetic agents; PSM: propensity score match.

Univariate logistic regression analysis was adopted to recognize factors that were statistically significant between CAC < 100 and ≥100. Among all the clinical characteristics, we identified eGFR (*P* < 0.001), metformin (*P* = 0.018), statins (*P* = 0.009), and antiplatelet drug prescription (*P* = 0.005) as predictive factors. Since combinations of medication might impact the outcome, we also took the number of nonmetformin antidiabetic drugs into consideration. Those factors together with demographic characteristics including age, BMI, male gender, smoking, and duration of diabetes and hypertension were reevaluated in a multivariable logistic regression analysis. The result indicated that metformin was still significantly associated with CAC severity (OR [95% CI] = 0.58 [0.35–0.96]; *P* = 0.035) (Figure 3, Supplemental Table 3). The association between metformin and CAC scores was consistent after PSM (OR [95% CI] = 0.58 [0.34–0.99]; *P* = 0.048, Figure 3, Supplemental Table 3).

### 3.4. Association between Metformin and CAC Severity in Smokers or Never-Smokers

Cigarette smoking was positively related to CAC scores (after PSM, OR [95% CI] = 2.08 [1.01-4.24]; *P* = 0.046). The interaction between smoking and metformin use was not significant (*P* = 0.371). Since smoking was the only modifiable factor that was associated with CAC scores, we decided to take a further look at this factor. One hundred and thirty-five subjects with a history of cigarette smoking were included. Notably, in our study, all smokers were male. In order to rule out gender difference, we first did a sensitivity analysis by looking at the association between metformin and CAC scores in both genders. A CAC score = 100 was set as the cutoff point in the analysis. After adjustment of the predictive factors and demographic characteristics, metformin was not related to CAC in male (OR [95% CI] = 0.64 [0.34–1.20]; *P* = 0.162) and female (OR [95% CI] = 0.42 [0.17–1.03]; *P* = 0.059) subgroups.

**Figure 4:**
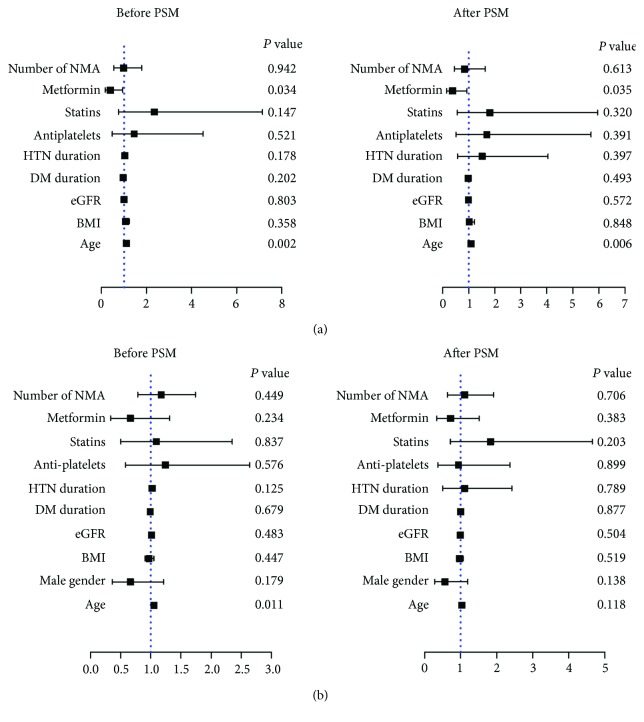
The relationship of CAC scores and metformin usage in smokers and never-smokers. Multivariable logistic regression revealed a significant association between metformin and CAC scores in male smokers before (OR [95% CI] = 0.40 [0.17–0.94]; *P* = 0.034) and after propensity score matching (PSM, OR [95% CI] = 0.38 [0.16–0.93]; *P* = 0.035) (a), but the association was not observed in never-smokers (OR [95% CI] = 0.72 [0.34–1.51]; *P* = 0.383) (b). BMI: body mass index; DM: diabetes mellitus; HTN: hypertension; eGFR: estimated glomerular filtration rate; NMA: nonmetformin antidiabetic agents.

We proceeded to perform subgroup analysis in subjects with or without a history of smoking. Multivariable logistic regression revealed a significant association between metformin and CAC scores in smokers (OR [95% CI] = 0.38 [0.16–0.93]; *P* = 0.035) (Figure 4(a), Supplemental Table 4), but the association diminished in never-smokers (OR [95% CI] = 0.72 [0.34–1.51]; *P* = 0.383) (Figure 4(b), Supplemental Table 5).

## 4. Discussion

In the present study, we investigated the association of metformin on CAC severity in T2DM populations. The major finding is that metformin is linked to a lower level of CAC scores in T2DM patients, especially in those with a history of cigarette smoking.

Metformin has been established for its unshakeable status in T2DM therapy as it reduces the morbidity and mortality of macrovascular complications [14]. Long-term metformin therapy is related to a 33% reduction in myocardial infarction [15]. In the CAMERA study, investigators also explored the effect of metformin on nondiabetic CAD. Even though metformin reduced the HbA1c level and improved insulin resistance, there was no difference in carotid intima-media thickness (cIMT) progression between the metformin and placebo groups [16]. However, the result was confounded by the small sample size and the use of statins, which made the window of opportunity for cIMT improvement rather small. We believe that the undergoing large randomized clinical trial GLINT might bring good news on the use of metformin in CAD patients without T2DM.

So far, the mechanism of metformin in CAD still needs to be explored. A randomized placebo-controlled trial conducted in 50 HIV-infected subjects with metabolic syndrome demonstrated that metformin reduces CAC progression [17], but the validity of the result in general diabetic population was hampered by the small sample size and HIV infection status in the study. Moreover, a recent study indicated that metformin treatment had protective effect on coronary atherosclerosis in male prediabetic population, but the result was inconsistent in female subjects [6]. However, in the present study, we found that there was no gender difference in the association between metformin and CAC. This might be explained by the difference in participants' age. In the former study, the average of female subjects was 49.1 ± 9.3, including 38% premenopausal, while the average age of female was 69.3 ± 9.8 in our study. It would be interesting to further investigate the association of metformin and CAC in premenopausal diabetic patients.

As mentioned, the current antidiabetic regimen was defined as drugs that were taken regularly without modification in prescription for at least 3 months. It was of interest that metformin rather than other antidiabetic agents was associated with lower CAC scores in our study. Since we did not have the information of antidiabetic medications they received in the past, subjects who discontinued metformin before the study gave rise to time-related bias. Yet, this bias tightened the association between metformin and CAC scores. It was because some patients in the nonmetformin group had metformin before the study, which would weaken the difference in the CAC scores between the metformin and nonmetformin groups in the study. A number of clinical studies had shown that metformin had cardiovascular protective effects independent of glucose-lowering effects [6, 18]. Since the CAC score is a well-accepted risk factor for cardiovascular events, the preventive effect of metformin on CAD, may at least partially, attributes to its role in reducing CAC.

The mechanisms by which metformin might result in lower CAC are still not fully understood. Our group revealed that metformin prevents atherosclerotic calcification via the activation of AMPK and subsequent Runx2 degradation [10]. Cao, et al. reported that metformin upregulated endothelial nitric oxide synthase (eNOS) in vascular smooth muscle cells to prevent calcification [9]. However, other mechanisms may also be involved, such as proautophagy, antioxidative stress, and anti-inflammation via both AMPK-dependent and AMPK-independent pathways [19].

It is noteworthy that metformin is profoundly linked with lower CAC scores in smoking subjects in the light of our study. Cigarette smoking is a well-established risk factor that is modifiable for CAD. The Heinz Nixdorf Recall study reported that smoking was also positively associated with CAC scores [20]. In another large retrospective cohort study in T2DM patients, researchers found that metformin is associated with lower risk of CAD and mortality in current and former smokers [21]. Smoking releases numerous toxic chemicals and free radicals, exposing the coronary artery to excess oxidative stress, while vascular calcification is also attributed to oxidative stress [22]. Metformin may exhibit its beneficial role in CAC due to its antioxidative stress properties [23] as well as its role in upregulating some components of the antioxidant defense system [24]. Nevertheless, the specific underlying mechanism is yet to be elucidated.

The limitations of our study include (1) the cross-sectional design, (2) involvement of relatively small numbers of subjects, (3) the missing data of HbA1c that hampered the interpretation of blood glucose control over the CAC severity, and (4) inability to exclude other interfering factors like lifestyle intervention. A prospective randomized controlled trial is needed to overcome these limitations. It would be interesting to examine the type and dose of antidiabetic medications, the CAC scores, and if possible, the change in the CAC scores.

To sum up, our results reveal that metformin prescription rather than other antidiabetic agents is negatively and independently associated with CAC severity in T2DM patients. We also witnessed a markedly negative association of metformin and CAC scores in smokers. The findings emphasize the use of metformin in all T2DM populations, especially those patients with a history of smoking.

## Figures and Tables

**Table 1:** The characteristics of included patients according to metformin usage.

Variables	Metformin usage (before PSM)			Metformin usage (after PSM)		
	Metformin (*n* = 150)	Nonmetformin (*n* = 219)	*P* value	Metformin (*n* = 150)	Nonmetformin (*n* = 138)	*P* value
Age	65.04 ± 9.47	70.03 ± 10.12	<0.001	65.04 ± 9.47	66.91 ± 9.69	0.100
BMI	25.20 ± 3.36	24.18 ± 3.52	0.005	25.20 ± 3.36	24.69 ± 3.41	0.206
Male gender	96 (64.00%)	132 (60.27%)	0.469	96 (64.00%)	85 (61.59%)	0.673
Smoking	61 (40.67%)	74 (33.79%)	0.178	61 (40.67%)	52 (37.68%)	0.604
DM duration	10.00 (4.75, 15.00)	8.00 (3.00, 15.00)	0.119	10.00 (4.75, 15.00)	7.00 (3.00, 15.00)	0.039
HTN	93 (62.00%)	163 (74.43%)	0.011	93 (62.00%)	98 (71.01%)	0.106
HTN duration	6.00 (0.00, 15.00)	10.00 (0.00, 20.00)	0.605	6.00 (0.00, 15.00)	5.25 (0.00, 15.00)	0.513
HbA1c (%)	7.50 (6.70, 8.15)	7.30 (6.78, 8.43)	0.990	7.50 (6.70, 8.15)	7.30 (6.80, 8.48)	0.988
eGFR (mL/min/1.73 m^2^)	96.53 (90.49, 105.50)	92.02 (81.63, 99.23)	<0.001	96.53 (90.49, 105.50)	94.82 (85.60, 101.10)	0.037
FBG (mmol/L)	6.64 (5.52, 8.57)	7.11 (5.76, 8.70)	0.480	6.64 (5.52, 8.57)	7.04 (5.66, 8.94)	0.395
Ca (mmol/L)	2.28 ± 0.16	2.25 ± 0.12	0.020	2.29 ± 0.16	2.26 ± 0.13	0.098
P (mmol/L)	1.15 ± 0.19	1.13 ± 0.20	0.429	1.15 ± 0.19	1.13 ± 0.20	0.361
LDL-C (mmol/L)	2.25 (1.61, 2.75)	2.30 (1.72, 2.89)	0.141	2.25 (1.61, 2.75)	2.48 (1.75, 2.99)	0.171
HDL-C (mmol/L)	1.05 (0.89, 1.28)	1.09 (0.91, 1.27)	0.835	1.05 (0.89, 1.28)	1.13 (0.93, 1.29)	0.153
TG (mmol/L)	1.46 (1.01, 2.15)	1.39 (0.98, 1.89)	0.219	1.46 (1.01, 2.15)	1.56 (0.99, 1.92)	0.900
Sulfonylureas	50 (33.33%)	86 (39.27%)	0.246	50 (33.33%)	56 (40.58%)	0.203
Glinides	28 (18.67%)	27 (12.33%)	0.093	28 (18.67%)	20 (14.49%)	0.429
GI	49 (32.67%)	100 (45.66%)	0.012	49 (32.67%)	53 (38.41%)	0.309
TZD	4 (2.67%)	12 (5.48%)	0.193	4 (2.67%)	8 (5.80%)	0.302
DPP4 inhibitor	2 (1.33%)	5 (2.28%)	0.511	2 (1.33%)	2 (1.45%)	0.675
Insulin	26 (17.33%)	54 (24.65%)	0.094	26 (17.33%)	31 (24.65%)	0.345
Statins	54 (36.00%)	62 (28.31%)	0.118	54 (36.00%)	43 (22.46%)	0.385
Antiplatelets	47 (31.33%)	70 (31.96%)	0.898	47 (31.33%)	43 (31.16%)	0.975
CAC scores	8.05 (0, 124.6)	61.6 (0, 319.6)	0.005	8.05 (0, 124.6)	37.00 (0.00, 220.50)	0.097

BMI: body mass index; DM: diabetes mellitus; GI: glucosidase inhibitors; HTN: hypertension; eGFR: estimated glomerular filtration rate; FBG: fasting blood glucose; Ca: calcium; P: phosphorus; LDL-C: low-density lipoprotein cholesterol; HDL-C: high-density lipoprotein cholesterol; TG: triglyceride; DPP4: dipeptidyl peptidase-4; TZD: thiazolidinediones; PSM: propensity score match.

**Table 2:** The characteristics of included patients according to CAC scores.

Variables	CAC scores (before PSM)			CAC scores (after PSM)		
	Score < 100 (*n* = 226)	Score ≥ 100 (*n* = 143)	*P* value	Score < 100 (*n* = 187)	Score ≥ 100 (*n* = 101)	*P* value
Age	65.65 ± 9.65	71.73 ± 9.83	<0.001	64.15 ± 9.25	69.24 ± 9.41	<0.001
BMI	24.57 ± 3.47	24.63 ± 3.53	0.886	24.96 ± 3.47	24.18 ± 3.52	0.987
Male gender	143 (63.27%)	85 (59.44)	0.460	119 (63.64%)	62 (61.39%)	0.706
Smoking	77 (34.07%)	58 (40.56)	0.207	68 (36.36%)	45 (44.55%)	0.174
DM duration	9.50 (4.00, 5.00)	10.00 (3.00,15.00)	0.814	9.00 (4.00, 15.00)	10.00 (3.00, 15.00)	0.993
HTN	149 (65.93%)	107 (74.83)	0.071	119 (63.64%)	72 (71.29%)	0.190
HTN duration	5.00 (0.00, 15.00)	10.00 (0.00, 20.00)	0.002	5.00 (0.00, 15.00)	8.00 (0.00, 15.00)	0.087
HbA1c (%)	7.4 (6.7, 8.3)	7.4 (6.8, 8.2)	0.790	7.40 (6.70-8.25)	7.45 (6.90-8.20)	0.456
eGFR (mL/min/1.73 m^2^)	95.70 (88.02, 104.88)	91.75 (81.47, 98.46)	<0.001	97.40 (89.74, 105.80)	92.46 (85.55, 99.03)	<0.001
FBG (mmol/L)	7.04 (5.91, 8.90)	6.82 (5.44, 8.39)	0.220	7.00 (5.93-8.94)	6.82 (5.38-8.35)	0.205
Ca (mmol/L)	2.26 ± 0.13	2.26 ± 0.16	0.963	2.27 ± 0.12	2.27 ± 0.18	0.924
P (mmol/L)	1.13 ± 0.19	1.16 ± 0.21	0.151	1.13 ± 0.19	1.15 ± 0.21	0.399
LDL-C (mmol/L)	2.27 (1.70, 2.85)	2.25 (1.60, 2.82)	0.603	2.23 (1.71-2.88)	2.27 (1.58-2.97)	0.848
HDL-C (mmol/L)	1.09 (0.81, 1.28)	1.03 (0.89, 1.27)	0.267	1.11 (0.92-1.29)	1.03 (0.88-1.27)	0.158
TG (mmol/L)	1.41 (0.99, 2.04)	1.39 (0.97, 1.88)	0.497	1.50 (1.00-2.06)	1.56 (0.98-2.00)	0.995
Metformin	107 (47.34%)	43 (30.07%)	0.001	107 (57.22%)	43 (42.57%)	0.018
Sulfonylureas	82 (36.28%)	54 (37.76%)	0.774	68 (36.36%)	38 (37.62%)	0.832
Glinides	33 (14.60%)	22 (15.38%)	0.837	31 (16.58%)	17 (16.83%)	0.956
GI	85 (37.61%)	64 (44.76%)	0.173	65 (34.76%)	37 (36.63%)	0.751
TZD	10 (4.42%)	6 (4.29%)	0.916	7 (3.74%)	5 (4.95%)	0.857
DPP4 inhibitor	5 (2.21%)	2 (1.40%)	0.711	4 (2.14%)	0 (0.00%)	0.341
Insulin	47 (20.80%)	33 (23.07%)	0.605	36 (19.25%)	21 (20.79%)	0.754
Statins	62 (27.43%)	54 (37.76%)	0.037	53 (28.34%)	44 (43.56%)	0.009
Antiplatelets	59 (26.11%)	58 (40.56%)	0.004	48 (25.67%)	42 (41.58%)	0.005

BMI: body mass index; DM: diabetes mellitus; GI: glucosidase inhibitors; HTN: hypertension; eGFR: estimated glomerular filtration rate; FBG: fasting blood glucose; Ca: calcium; P: phosphorus; LDL-C: low-density lipoprotein cholesterol; HDL-C: high-density lipoprotein cholesterol; TG: triglyceride; DPP4: dipeptidyl peptidase-4; TZD: thiazolidinediones; PSM: propensity score match.

## Data Availability

The clinical data used to support the findings of this study are restricted by the Ethics Committee of the Second Affiliated Hospital, Zhejiang University School of Medicine, in order to protect patient privacy. Data are available from the corresponding author for researchers who meet the criteria for access to confidential data.

## Supplementary Materials

Supplementary Materials Supplemental Figure 1: the flow chart of the patient inclusion and exclusion. Supplemental Table 1: the characteristics of included patients according to CAC scores. Supplemental Table 2: the association between metformin and other antidiabetic medicine and CAC scores. Supplemental Table 3: the independent association of metformin and other factors with CAC scores. Supplemental Table 4: the subgroup analysis in patients with a history of smoking. Supplemental Table 5: the subgroup analysis in patients without a history of smoking.
