# Supplementary material for: Association between Metformin Use and Coronary Artery Calcification in Type 2 Diabetic Patients
Source: J Diabetes Res. 2019 May 5;2019:9484717. doi: 10.1155/2019/9484717 (PMC6525896; doi:10.1155/2019/9484717)
Supplement: Supplementary Materials — Supplemental Figure 1: the flow chart of the patient inclusion and exclusion. Supplemental Table 1: the characteristics of included patients according to CAC scores. Supplemental Table 2: the association between metformin and other antidiabetic medicine and CAC scores. Supplemental Table 3: the independent association of metformin and other factors with CAC scores. Supplemental Table 4: the subgroup analysis in patients with a history of smoking. Supplemental Table 5: the subgroup analysis in patients without a history of smoking. [file 9484717.f1.pdf]

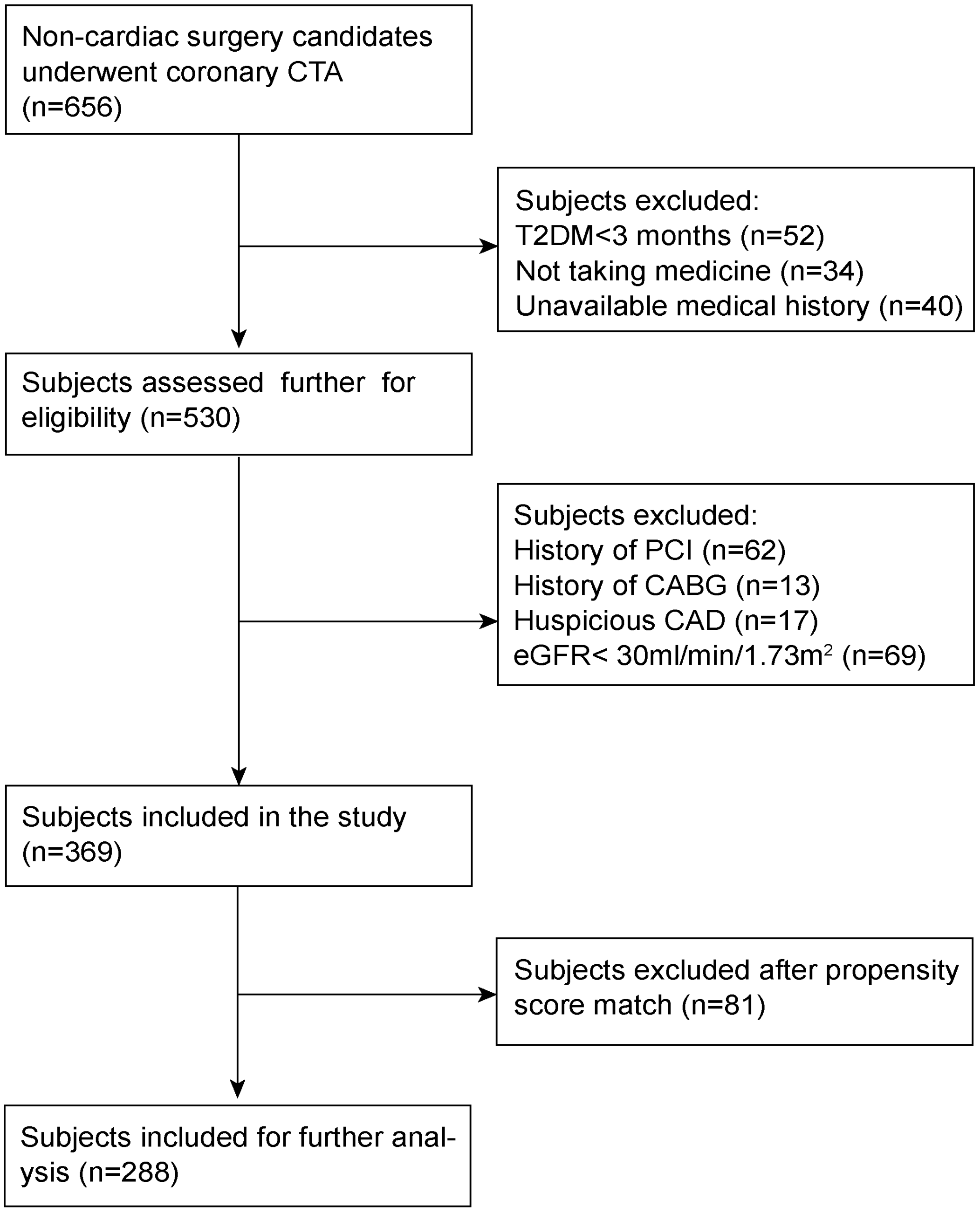

Supplemental Figure 1. The flow chart of the patients inclusion and exclusion.

Supplemental Table 1. The characteristics of included patients according to CAC scores

	CAC = 0 (n = 146)	0 < CAC < 100 (n = 79)	CAC ≥ 100 (n = 143)	P value
**Age**	64.08 ± 9.53	68.23 ± 8.95	71.73 ± 9.83	<0.001
**BMI**	24.49 ± 3.42	24.77 ± 3.56	24.63 ± 3.53	0.838
**Male gender**	85 (58.22%)	57 (72.15%)	85 (59.44%)	0.095
**Smoking**	46 (31.51%)	31 (39.24%)	58 (40.56%)	0.243
**DM duration**	8.0 (5.0, 15.0)	10.0 (3.0, 15.0)	10.0 (3.0, 15.0)	0.760
**HTN**	102 (69.86%)	47 (59.49%)	107 (74.83%)	0.059
**HTN duration**	8.0 (0.0, 15.0)	4.0 (0.0, 10.0)	10.0 (0.0, 20.0)	0.003
**HbA1c (%)**	7.3 (6.6, 8.1)	7.7 (7.1, 9.1)	7.4 (6.8, 8.2)	0.051
**eGFR (mL/min/1.73m^2)**	95.3 (88.39, 102.8)	96.84 (87.40, 105.82)	91.75 (81.47, 98.46)	0.001
**FBG (mmol/L)**	6.99 (5.61, 8.98)	7.12 (6.06, 8.71)	6.82 (5.44, 8.39)	0.309
**Ca (mmol/L)**	2.27 ± 0.12	2.25 ± 0.14	2.26 ± 0.16	0.506
**P (mmol/L)**	1.13 ± 0.20	1.12 ± 0.16	1.16 ± 0.21	0.368
**LDL-C (mmol/L)**	2.22 (1.65, 2.87)	2.37 (2.00, 2.84)	2.25 (1.61, 2.77)	0.498
**HDL-C (mmol/L)**	1.11 (0.92, 1.32)	1.03 (0.90,1.22)	1.03 (0.89, 1.27)	0.119
**TG (mmol/L)**	1.40 (0.99, 2.02)	1.51 (1.00, 2.53)	1.39 (0.97, 1.88)	0.393
**Metformin**	68 (46.58%)	39 (49.37%)	43 (30.07%)	0.004
**Sulfonylureas**	57 (39.04%)	25 (31.65%)	54 (37.76%)	0.530
**Glinides**	22 (15.07%)	11 (13.92%)	22 (15.38%)	0.957
**GI**	46 (31.51%)	39 (49.37%)	64 (44.76%)	0.014
**TZD**	8 (5.48%)	2 (2.53%)	6 (4.20%)	0.582
**DPP4 inhibitor**	3 (2.05%)	2 (2.53%)	2 (1.40%)	0.888
**Insulin**	26 (17.81%)	21 (26.58%)	33 (23.08%)	0.277
**Statins**	39 (26.71%)	22 (27.85%)	54 (37.76%)	0.098

BMI, body mass index; DM, diabetes mellitus; GI，glucosidase inhibitors； HTN,

hypertension; eGFR, estimated glomerular filtration rate; FBG, fasting blood glucose; Ca, calcium; P, phosphorus; LDL-C, low-density lipoprotein cholesterol; HDL-C, high-density lipoprotein cholesterol; TG, triglyceride; DPP4, dipeptidyl peptidase-4; TZD, thiazolidinediones；

Supplemental Table 2. The association between metformin or other anti-diabetic medicine and CAC scores

	Before PSM (n = 369)			After PSM (n = 288)		
**Variables**	**OR**	**95%CI**	**P value**	**OR**	**95%CI**	**P value**
**Metformin**	0.48	0.31 - 0.75	0.001	0.55	0.34 - 0.90	0.018
**Sulfonylurea**	1.07	0.69 - 1.64	0.774	1.06	0.64 - 1.74	0.832
**Glinide**	1.06	0.59 - 1.91	0.837	1.02	0.53 - 1.95	0.956
**GI**	1.34	0.88 - 2.06	0.173	1.09	0.66 - 1.80	0.751
**TZD**	0.95	0.34 - 2.66	0.916	1.34	0.41 - 4.33	0.626
**DPP4 inhibitor**	0.63	0.12 - 3.28	0.580	N/A	N/A	N/A
**Insulin**	1.14	0.69 - 1.89	0.605	1.10	0.60 - 2.01	0.754

GI, glucosidase inhibitors; PSM, propensity score match; TZD, thiazolidinediones; DPP4, dipeptidyl peptidase-4; PSM, propensity score match. DPP4 inhibitor user was 0 among subjects with CAC ≥ 100 after PSM.

Supplemental Table 3. The independent association of metformin and other factors with CAC scores

	Before PSM (n = 369)			After PSM (n = 288)		
**Variables**	**OR**	**95%CI**	**P value**	**OR**	**95%CI**	**P value**
**Age**	1.06	1.03 - 1.10	<0.001	1.05	1.02 - 1.09	0.003
**Male gender**	0.69	0.37 - 1.29	0.245	0.65	0.31 - 1.35	0.246
**BMI**	0.99	0.93 - 1.06	0.828	0.97	0.90 - 1.05	0.506
**eGFR**	1.00	0.99 - 1.02	0.692	0.99	0.97 - 1.01	0.443
**DM duration**	0.99	0.95 - 1.02	0.398	0.99	0.96 - 1.03	0.612
**HTN duration**	1.02	1.00 - 1.05	0.049	1.01	0.99 - 1.04	0.286
**Smoking**	2.05	1.11 - 3.79	0.022	2.08	1.01 - 4.24	0.046
**Anti-platelets**	1.41	0.76 - 2.59	0.276	1.27	0.62 - 2.57	0.512
**Statins**	1.33	0.72 - 2.45	0.357	1.72	0.86 - 3.47	0.128
**Metformin**	0.58	0.35 - 0.96	0.035	0.58	0.34 - 0.99	0.048
**Number of NMA**	1.07	0.77 - 1.48	0.697	0.97	0.65 - 1.46	0.885

BMI, body mass index; DM, diabetes mellitus; HTN, hypertension; eGFR, estimated glomerular filtration rate; NMA, non-metformin anti-diabetic agents; PSM, propensity score match.

Supplemental Table 4. The subgroup analysis in patients with a history of smoking

	Before PSM (n = 135)			After PSM (n = 113)		
**Variables**	**OR**	**95%CI**	**P value**	**OR**	**95%CI**	**P value**
**Age**	1.10	1.04 - 1.17	0.002	1.09	1.03 - 1.17	0.006
**BMI**	1.07	0.93 - 1.23	0.358	1.02	0.87 - 1.19	0.848
**eGFR**	1.00	0.96 - 1.03	0.803	0.99	0.96 - 1.03	0.572
**DM duration**	0.96	0.91 - 1.02	0.202	0.98	0.92 - 1.04	0.493
**HTN duration**	1.03	0.99 - 1.07	0.178	1.52	0.57 - 4.05	0.397
**Anti-platelets**	1.45	0.47 - 4.51	0.521	1.70	0.51 - 5.70	0.391
**Statins**	2.35	0.75 - 7.15	0.147	1.82	0.56 - 5.95	0.320
**Metformin**	0.40	0.17 - 0.94	0.034	0.38	0.16 - 0.93	0.035
**Number of NMA**	0.98	0.54 - 1.78	0.942	0.84	0.44 - 1.63	0.613

BMI, body mass index; DM, diabetes mellitus; HTN, hypertension; eGFR, estimated glomerular filtration rate; NMA, non-metformin anti-diabetic agents; PSM, propensity score match.

Supplemental Table 5. The subgroup analysis in patients without a history of smoking

	Before PSM (n = 234)			After PSM (n = 175)		
**Variables**	**OR**	**95%CI**	**P value**	**OR**	**95%CI**	**P value**
**Age**	1.05	1.01 - 1.09	0.011	1.03	0.99 - 1.08	0.118
**Male gender**	0.66	0.36 - 1.21	0.179	0.57	0.28 - 1.20	0.138
**BMI**	0.97	0.90 - 1.05	0.447	0.97	0.88 - 1.07	0.519
**eGFR**	1.01	0.99 - 1.03	0.483	0.99	0.97 - 1.02	0.504
**DM duration**	0.99	0.95 - 1.04	0.679	1.00	0.95 - 1.05	0.877
**HTN duration**	1.02	0.99 - 1.05	0.125	1.11	0.51 - 2.42	0.789
**Anti-platelets**	1.24	0.58 - 2.64	0.576	0.94	0.37 - 2.37	0.899
**Statins**	1.09	0.50 - 2.35	0.837	1.83	0.72 - 4.65	0.203
**Metformin**	0.66	0.33 - 1.31	0.234	0.72	0.34 - 1.51	0.383
**Number of NMA**	1.17	0.78 - 1.74	0.449	1.11	0.64 - 1.91	0.706

BMI, body mass index; DM, diabetes mellitus; HTN, hypertension; eGFR, estimated glomerular filtration rate; NMA, non-metformin anti-diabetic agents; PSM, propensity score match.
